# A value-oriented psychological contract: Generational differences amidst a global pandemic

**DOI:** 10.3389/fpsyg.2022.921184

**Published:** 2022-07-25

**Authors:** Alda Deas, Melinde Coetzee

**Affiliations:** ^1^Department of Human Resource Management, University of South Africa, Pretoria, South Africa; ^2^Department of Industrial and Organizational Psychology, University of South Africa, Pretoria, South Africa

**Keywords:** Psychological Contract Inputs-Outcomes Inventory, equity theory, COVID-19, employe input obligations, employe organizational outcome expectations, psychological contract, generational differences, digital worker

## Abstract

The COVID-19 pandemic has changed the landscape of working conditions world-wide, fast tracking the reality of the digital-driven workplace. Concepts such as remote working, working-from-home and hybrid working models are now considered as the “new normal.” Employes are expected to advance, flourish and survive in this digitally connected landscape. Different age and generational groups may experience this new organizational landscape differently and may expect different organizational outcomes in exchange for their inputs. Accordingly, the study investigated differences regarding the value-oriented psychological contract expectations of employes from different generational groups. An ANOVA test for significant mean differences and a *post hoc* test for multiple comparisons were conducted on a sample of (*N* = 293) employes in the services industry in Southern Africa (85%) and other European countries (15%). The observed generational cohort differences regarding value-orientated psychological contract expectations for job characteristics and work-life balance could be utilized to develop interventions and strategies to promote retention of employes in the post-pandemic digital-orientated workplace.

## Introduction

The Covid-19 pandemic has resulted in significant economic and social challenges for nations globally (Potgieter, 2021) and transformed the way in which we worked, studied, traveled and lived in general (Lopez and Fuiks, 2021). The world-wide shutdown that was implemented in order to control the pandemic, resulted in various challenges for the employment relationship (Kniffen et al., 2021) as it disrupted and transformed workplace policies and practices significantly (Lee, 2021). Covid-19 has also placed a renewed emphasis on the implementation of information and communication technologies (ICT), that has influenced the way in which we manage human capital (Bester and Bester, 2021; Potgieter, 2021). As Stofberg et al. (2021) noted, the digital revolution has transformed the workplace and organizations had to adapt in order to survive this tsunami called Industry 4.0. Managing employes and their expectations is therefore of utmost importance.

Recent research has also indicated that alternative work arrangements, teamwork through virtual platforms and contingent work arrangements augmented by the Covid-19 pandemic and the digital revolution have resulted in the birth of the value-oriented psychological contract for the digital worker (Coetzee, 2021; Veldsman and Van Aarde, 2021; Deas and Coetzee, 2022). However, employers should be mindful that employes from different generational groups may respond differently to the changes brought on by the digital revolution and the Covid-19 pandemic (Shanmugam, 2016; Dhliwayo, 2021). Conversations concerning value-oriented generational differences are commonplace for organizational science and practice (Rudolph et al., 2021) especially when considering that traditional employment relationships are rapidly changing to non-traditional employment relationships and part-time, fixed-term work arrangements (Alcover et al., 2017; Kutaula et al., 2020). Accordingly, the objective of this study was to explore differences regarding the value-oriented psychological contract expectations of employes from different age-grouped generational cohorts. We hypothesized that employes from various generational groups will differ in terms of their perceptions of the value-oriented psychological contract. The concept of the psychological contract has been studied extensively; however, the concept of the value-orientated psychological contract is still under-researched (Coetzee et al., 2022; Deas and Coetzee, 2022). The results of this article will therefore contribute to new knowledge on this concept from a generational perspective.

## The value-oriented psychological contract

The psychological contract represents an essential part of the employment relationship and is mainly based on the power of perception (Veldsman and Van Aarde, 2021; Perkins et al., 2022). Argyris (1960) conceptualized the psychological contract as the perception of mutual expectations underlining the exchange agreement in the employment relationship. Drawing from Adams (1965) equity theory, the value-oriented psychological contract refers to employes’ perception of equity in terms of the organizational obligated outcomes in exchange for their obligated inputs. It is argued that employes will be satisfied with their employment relationship if they perceive that there is an equitable balance between what they receive from the organization in return for what they give to the organization (Payne et al., 2015; Coetzee et al., 2022). Expectations normally include aspects such as compensation and benefits, training opportunities and skills development, and job characteristics (Nayak et al., 2021).

Research on the psychological contract typically concentrates on either one of the two predominant themes, namely content-based or evaluation-based psychological contract expectations (Kutaula et al., 2020). Various researchers have emphasized the necessity to examine the contents of the psychological contract; however these studies are more concentrated on the traditional employes’ perceived obligations (Rousseau, 1989; Karani et al., 2021). Generally, the content-based approach focus on the transactional and relational content-elements of the psychological contract (Kutaula et al., 2020). According to Coetzee (2021), the traditional transactional psychological contract generally refers to specific, short-term and monetary benefits, based on financial exchange agreements, whereas a relational psychological contract refers to open-ended or extended employment agreements. Evaluation-based psychological contract research, on the other hand, is focused on determining the fulfilment, or breach of these psychological contract content-elements (Santos et al., 2019).

Against this backdrop, Deas (2021) conceptualized four dimensions for the value-oriented psychological contract, namely employe obligated inputs, organizational obligated outcomes, employe obligated inputs delivered and psychological contract fulfilment. Employe obligated inputs refers to both task obligatory aspects (e.g., meeting task requirements and acting ethically and honestly) and attitudinal obligatory aspects (e.g., being engaged and loyal toward the organizational brand, vision and mission) (Coetzee et al., 2022; Deas and Coetzee, 2022). Organizational obligated outcomes include aspects such as organizational culture, career development opportunities, work-life balance, rewards, job characteristics and relationships (Deas and Coetzee, 2022). The employe obligated inputs delivered dimension and the psychological contract fulfilment dimension act as perceived equity ratio measures (Coetzee et al., 2022).

## Generational differences in terms of work values

Based on the generational cohort theory, people who grew up and experienced the same historical events during their emotional developmental years will belong to the same generational cohort (Ryder, 1965; Jung et al., 2021). Research has distinguished between four generational (age-grouped) cohorts currently in the workplace ranging from the Baby Boomers (1946–1965), Generation X (1966–1980), Generation Y (1981–1994) and the final generational cohort joining the workforce, Generation Z (1995 and after) (Chaney et al., 2017; Lissitsa and Kol, 2021). It is widely believed that different generational cohorts bring different values, attitudes and behaviors to the workplace (Gabrielova and Buchko, 2021) and these different values, attitudes and behaviors should be understood in order to successfully manage employes from different generational cohorts (Kirchmayer and Fratričová, 2020). Both researchers and practitioners are apprehensive about the impact of generational differences on the workplace and the issues these value-based differences can create for human resource practitioners (Stark and Poppler, 2018). As a result, the impact of generational differences on the employment relationship has been studied in terms of job-related aspects and work-related values (Goh and Jie, 2019; Jung et al., 2021). However, little research has focused on the generational effect on the psychological contract (Lub et al., 2016; Magni and Manzoni, 2020). Magni and Manzoni (2020) postulate that, together with age, it is important to examine the psychological contract from a generational perspective as this might have a stronger impact on psychological contract expectations than merely examining it from an age perspective. Accordingly, the objective of the study was to investigate differences regarding the value-oriented psychological contract expectations of employes from different generational groups.

## Materials and methods

### Participants

Contemporary workers (*N* = 293) from human resource and financial services organizations across Southern Africa (85%) and various European countries (15%) were included in this study by means of a convenience sampling method. Demographics for this sample are mostly represented by the Black (63%) men (54%) from the Generation Z generational cohort (53%, ages between 26 and 40 years).

### Measuring instrument

The *Psychological Contract Input-Outcomes Inventory* (PCIOI) (Deas, 2021; Coetzee et al., 2022) is a 46-item multi-level, 5-point Likert-type (ranging from 1 = not at all to 5 = to a great extent) scale measuring four dimensions of the value-oriented psychological contract. The first dimension of this scale, the employe obligated inputs dimension (12-items), measures employes’ perceptions in terms of primary task performance obligations (e.g., “I feel obligated to provide inputs and ideas to execute tasks”) and secondary attitudinal obligations (e.g., “I feel obligated to fulfill the organization’s vision, mission and values”). The second dimension, the organizational outcomes dimension (29-items), measures employes’ perceptions of organizational outcomes, including organizational culture (e.g., “I expect equal treatment of all employes”), career development opportunities (e.g., “I expect to receive learning/coaching/mentoring on the job”), work-life balance (e.g., “I expect flexibility in terms of where and when I do my job”), rewards (e.g., “I expect job security”), relationships (e.g., “I expect opportunities for teamwork”), and job characteristics (e.g., “I expect innovative work challenges”).

The third dimension, the psychological contract fulfilment dimension (5-items), measures employes’ perceptions on the organizations’ fulfilment of employe expectations (e.g., “I feel the organization fulfilled my needs for autonomy and challenging job characteristics”). The final dimension, the employe obligated inputs delivered dimension (2-items), is based on a self-reflection on whether employes’ delivered on their primary tasks and secondary obligations toward the organization (e.g., “I feel I delivered on the primary employe inputs to the organization”). Deas and Coetzee (2022) provided evidence of the construct validity and internal consistency reliability for the four-dimensional scale in the South African context.

### Procedure

The online platform LinkedIn was used to invite participants to complete a voluntary, anonymous survey (LimeSurvey GmbH, 2020). Data obtained were transferred to a SPSS file for data analysis.

### Ethical considerations

Ethical clearance to conduct the research was obtained from the University of South Africa (ERC Ref#: 2020_CEMS/IOP_014). Participants were advised that participation was completely voluntary, anonymous, confidential and private. Informed consent was also attained in order to use the data for research purposes.

### Data analysis

Data were analyzed using IBM Corp (2020) SPSS Version 27 and SAS/STAT^®^ software version 9.4M5© (2017). Test for significant mean differences were conducted to determine the differences between age/generational cohorts and their perceptions in terms of the value-oriented psychological contract. ANOVA’s were used to determine the differences among the variables.

## Results

Table 1 indicates that the generational groups appeared to differ significantly in respect of the organizational obligated outcomes and employe obligated inputs delivered constructs. The Bonferonni’s test for multiple comparisons showed significant mean differences in terms of work-life balance for the 30 years and younger (Gen Z) (M = 3.83; SD = 0.91) versus the 46–55 years (Gen X) (M = 4.26; SD = 0.66; ω^2^ = 0.02; small practical effect) generational groups [*p* ≤ 0.05; C.I. = (−0.8128; −0.0350)].

**TABLE 1:** ANOVA (with *Post Hoc* Bonferonni Tests): Organizational obligated outcomes, employe inputs delivered and generational groups.

Variable	Age group	*N*	Mean	SD	ω ^2^	df	F	Sig	Source of significant difference between means	Mean differences 95% CI [LL;UL]
Organizational outcomes (work-life balance)	30 years and younger (Gen Z)	64	3.83	0.91	0.023	3	3.290	0.021*	46–55 years (Gen X)	−0.42* [−0.8128; −0.0350]
	31–45 years (Gen Y)	151	4.13	0.82						
	46–55 years (Gen X)	58	4.26	0.66					30 years and younger (Gen Z)	0.42* [0.0350;0.8128]
	56–65 years (Baby Boomers)	16	3.93	0.68						
Organizational outcomes (job characteristics)	30 years and younger (Gen Z)	64	4.33	0.71	0.021	3	3.042	0.029*	46–55 years (Gen X)	−0.29* [−0.5865; −0.0112]
	31–45 years (Gen Y)	151	4.39	0.58					46–55 years (Gen X)	−0.25* [−0.4910;−0.0007]
	46–55 years (Gen X)	58	4.63	0.49					30 years and younger (Gen Z) 31–45 years (Gen Y)	0.29* [0.0112;0.5865] 0.25* [0.0007;0.4910]
	56–65 years (Baby Boomers)	16	4.44	0.65						
Employe obligated inputs delivered	30 years and younger (Gen Z)	64	3.83	0.88	0.045	3	5.491	0.001***	31–45 years (Gen Y) 46–55 years (Gen X)	−0.44*** [−0.7427; −0.1441] −0.39* [−0.7598; −0.0323]
	31–45 years (Gen Y)	151	4.27	0.71					30 years and younger (Gen Z)	0.44*** [0.1441;0.7427]
	46–55 years (Gen X)	58	4.22	0.72					30 years and younger (Gen Z)	0.39* [0.0323;0.7598]
	56–65 years (Baby Boomers)	16	4.28	0.79						

Source: Authors’ own work.

***p ≤ 0.001, **p ≤ 0.01, **p ≤ 0.05.

CI, confidence interval. LL, lower level. UL, upper level.

The results indicated significant mean differences regarding job characteristics for the 30 years and younger (Gen Z) (M = 4.33; SD = 0.71) versus the 46–55 years (Gen X) (M = 4.63; SD = 0.49; ω^2^ = 0.02; small practical effect) generational groups {*p* ≤ 0.05; C.I. = [(0.0350;0.8128)]}, as well as the 31–45 years (Gen Y) (M = 4.39; SD = 0.58) vs. the 46–55 years (Gen X) (M = 4.63; SD = 0.49; ω^2^ = 0.02; small practical effect) generational groups {*p* ≤ 0.05; C.I. = [(0.0007;0.4910)]}.

In terms of the employe obligated inputs delivered construct, the results indicated significant mean differences for the 30 years and younger (M = 3.83; SD = 0.88) versus the 31–45 years (Gen Y) (M = 4.27; SD = 0.71; ω^2^ = 0.045; small practical effect) generational groups {*p* ≤ 0.001; C.I = [(−0.7427; −0.1441)]}, as well as for the 30 years and younger (M = 3.83; SD = 0.88) vs. the 46–55 years (Gen X) (M = 4.22; SD = 0.72) generational groups {*p* ≤ 0.05; C.I. = [(−0.7598; −0.0323)]}.

## Discussion

The current study set out to investigate whether age-grouped generational cohorts differ in terms of their value-oriented psychological contract expectations. More specifically, the findings suggest differences in terms of work-life balance and job characteristics expectations. According to Sánchez-Hernández et al. (2019), an important aspect for the younger generations is to combine work and family life in such a way as to create a strong work-life balance. Further to this, the younger the generation, the more value is placed on work-life balance and relaxation and less value is place on work ethic and the importance of work to an employe’s life (Lyons and Kuron, 2014; Brink and Zondag, 2019). Brink and Zondag (2019) also reported that the significance of flexible work-life policies increased across the generational cohorts. In terms of job characteristics, previous research has indicated that workers from different generational groups may react differently toward similar job characteristics (Kanfer and Ackerman, 2004; Zaniboni et al., 2013; Hernaus and Vokic, 2014). Vui-Yee and Paggy (2018) further assert that differences in age may impact on job-related aspects. According to findings from Stark and Poppler (2018), Baby boomers and Generation X employes indicated that they value a work that has meaning and affords a sense of achievement. Generation Z employes, on the other hand, value high-quality feedback and guidance (Zhang and Zhao, 2021). Job characteristics are regarded as a significant factor contributing to employe retention (Vui-Yee and Paggy, 2018). Accordingly, human resource practitioners should ensure that employes’ job characteristics are aligned with their values and expectations.

## Implications, limitations and directions for future research

The results of this study suggest important practical implications for work-life balance and job characteristics as important content-elements of the value-oriented psychological contract for different generational groups. The study corroborated that generational groups tend to differ regarding their work-life balance and job characteristics psychological contract expectations. Human resource practitioners may therefore adapt work-life balance policies in order to accommodate different age-grouped generational cohort values. Human resource practitioners should also focus on offering customized and individualized human resource practices that address the job characteristic needs of employes from different generational cohorts (Malik et al., 2020).

The limitations of this research suggest some insights for future research. The results of this cross-sectional study were largely restricted to employes from Southern Africa and cannot be generalized as such. Furthermore, all four generations were not equally sampled with the participants being predominantly from the Generation Y cohort. Also, participants were requested to complete a self-reported survey, therefore causal inferences are not possible. Future research could consider test-retest studies with a more equal representation of the generational cohorts across various occupational fields around the globe. Aside from these limitations, this study encourages new opportunities for research on the value-oriented psychological contract of employes, especially in the new digital work environment.

## Conclusion

This article contributed to the lack of and emerging body of knowledge on the value-oriented psychological contract. It also subsequently emphasized generational cohort differences in terms of organizational obligated outcome expectations for work-life balance and job characteristics. While the results of this empirical study may possibly be reinforced through further reproduction and investigation, it is believed that this article may stimulate further research and consideration in the measurement of employes’ value-oriented psychological contract through the Psychological Contract Inputs-Outcomes Inventory (PCIOI) in order to better understand the values and expectations of employes in the post-pandemic digital-revolutionized world of work.

## Data availability statement

The raw data supporting the conclusions of this article will be made available by the authors, without undue reservation.

## Ethics statement

The studies involving human participants were reviewed and approved by ERC Ref#: 2020_CEMS/IOP_014 University of South Africa. The patients/participants provided their written informed consent to participate in this study.

## Author contributions

All authors listed have made a substantial, direct, and intellectual contribution to the work, and approved it for publication.

## Conflict of interest

The authors declare that the research was conducted in the absence of any commercial or financial relationships that could be construed as a potential conflict of interest. The reviewers AR, SR, and handling editor declared their shared affiliation.

## Publisher’s note

All claims expressed in this article are solely those of the authors and do not necessarily represent those of their affiliated organizations, or those of the publisher, the editors and the reviewers. Any product that may be evaluated in this article, or claim that may be made by its manufacturer, is not guaranteed or endorsed by the publisher.
